# Emergency Surgical Management of Chemotherapy-Induced Tumor Rupture in a Patient with MYCN-Amplified Neuroblastoma: A Case Report

**DOI:** 10.70352/scrj.cr.25-0473

**Published:** 2025-11-08

**Authors:** Katsuhiro Ogawa, Satoshi Makita, Ami Utsunomiya, Hajime Asai, Hiroki Ishii, Daiki Kato, Yoichi Nakagawa, Kaito Hayashi, Shunya Takada, Aitaro Takimoto, Akihiro Yasui, Masamune Okamoto, Takahisa Tainaka, Chiyoe Shirota, Hiroo Uchida

**Affiliations:** Department of Pediatric Surgery, Nagoya University Graduate School of Medicine, Nagoya, Aichi, Japan

**Keywords:** neuroblastoma, tumor rupture, MYCN amplification, abdominal compartment syndrome, pediatric oncology

## Abstract

**INTRODUCTION:**

Tumor rupture with neuroblastoma is an uncommon but serious complication, particularly in high-risk cases involving MYCN amplification. When rupture occurs soon after induction chemotherapy is initiated, rapid deterioration and abdominal compartment syndrome (ACS) may develop. Early identification of high-risk patients and their readiness for surgical management are essential to optimizing outcomes.

**CASE PRESENTATION:**

A 4-year-old girl presented with a large left adrenal mass and elevated neuroblastoma markers. Imaging showed a heterogeneous 11 × 9.5 × 17-cm tumor encasing the renal hilum. Because of intratumoral hemorrhage, biopsy was deferred and induction chemotherapy was initiated. Seven days later, sudden abdominal distension and severe anemia developed. Contrast-enhanced CT confirmed intraperitoneal bleeding from the ruptured tumor. Transarterial embolization, including occlusion of the left renal artery, achieved temporary hemostasis; however, intra-abdominal pressure increased to 20 mmHg, thus meeting the ACS criteria. Emergency laparotomy revealed extensive hemorrhagic ascites and a ruptured tumor capsule. En bloc resection of the tumor and left nephrectomy were performed over 4 hours, and total blood loss of 2968 mL occurred. Histopathology confirmed MYCN-amplified neuroblastoma invading the adjacent renal parenchyma, which was classified as high risk by the International Neuroblastoma Risk Group. Postoperatively, the chylous ascites resolved by day 11, and multimodal therapy, including chemotherapy, autologous stem cell transplantation, proton beam radiotherapy, and anti-GD2 antibody therapy, was completed. Remission has been maintained for 2 years.

**CONCLUSIONS:**

Embolization alone may not prevent ACS in patients with MYCN-amplified neuroblastoma and chemotherapy-induced rupture. Prompt surgical resection can be life-saving when anatomically feasible. A pretreatment risk assessment, cautious initiation of chemotherapy, vigilant monitoring, and early surgical preparedness are critical for managing high-risk neuroblastoma.

## Abbreviations

ACS
: abdominal compartment syndrome
Cre
: creatinine
FFP
: fresh frozen plasma
RCC
: red cell concentrate
TAE
: transarterial embolization

## INTRODUCTION

Neuroblastoma is one of the most prevalent types of solid tumors in the pediatric population. Cases diagnosed at an advanced stage are generally treated using a multimodal approach that includes chemotherapy, surgical intervention, radiotherapy, and immunotherapy.^1)^ MYCN-amplified tumors are associated with a poor prognosis and require intensive therapeutic interventions.^2)^ Although tumor rupture during the initiation of induction chemotherapy is infrequent, it can result in severe complications, including massive intraperitoneal hemorrhage and abdominal compartment syndrome (ACS), particularly in cases characterized by a substantial tumor burden or vascular involvement.^3,4)^ Prompt identification and effective management of these complications are crucial to ensuring patient survival.^5)^

We present a rare case of high-risk MYCN-amplified neuroblastoma in a 4-year-old girl who developed tumor rupture and ACS soon after the initiation of induction chemotherapy that required emergency surgical intervention. Additionally, we examined the significance of risk assessments and therapeutic strategies to similar high-risk scenarios by reviewing the literature.

## CASE PRESENTATION

**Fig. 1:**
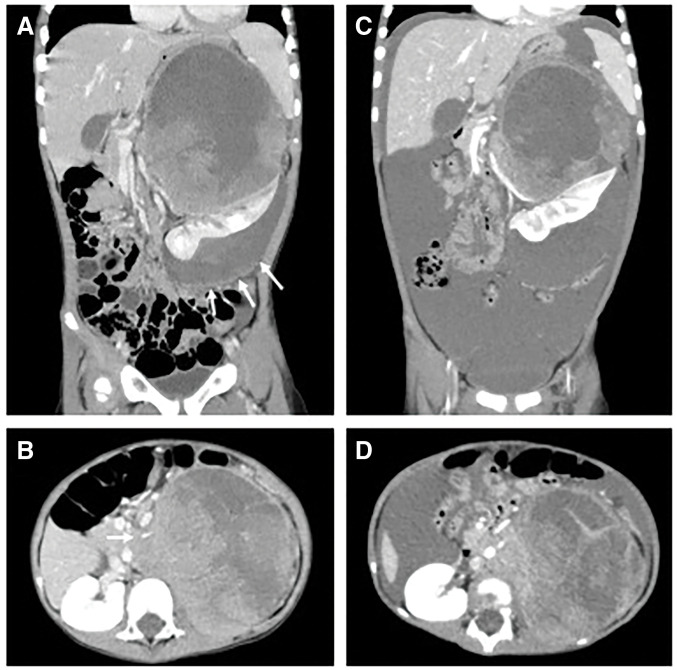
Contrast-enhanced abdominal CT findings. (**A**, **B**) Initial contrast-enhanced abdominal CT revealed a well-defined 17 × 11 × 9.5-cm heterogeneous mass compressing the left kidney. A low-density area lateral to the kidney encapsulated by a membrane suggestive of a hematoma was also identified (white arrows in **A**). The left renal artery and vein were encased between the tumor and enlarged para-aortic lymph nodes, consistent with IDRFs (white arrow in **B**). (**C**, **D**) Contrast-enhanced CT performed 7 days after the initiation of induction chemotherapy revealed increased ascites and findings suggestive of tumor rupture. IDRF, imaging-defined risk factor

**Fig. 2:**
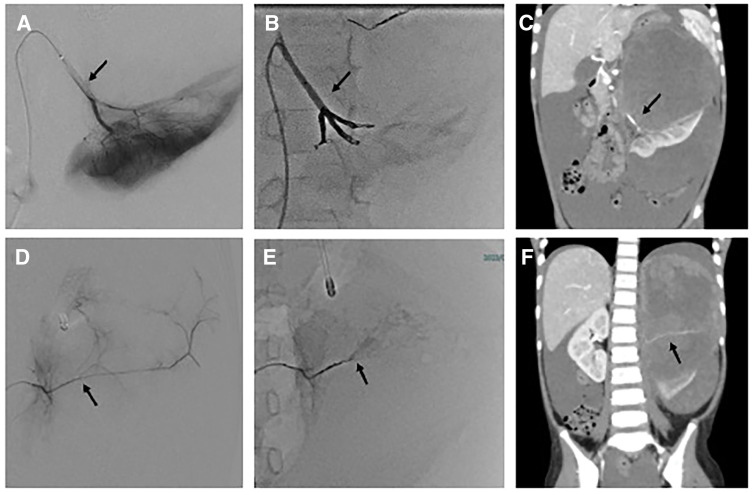
Angiography findings. (**A**–**C**) Selective angiography images of the left renal artery (arrows) before and after transarterial embolization. (**D**–**F**) Images obtained after selective angiography and embolization of the left adrenal artery (arrows), which was identified as a tumor-feeding vessel.

**Fig. 3:**
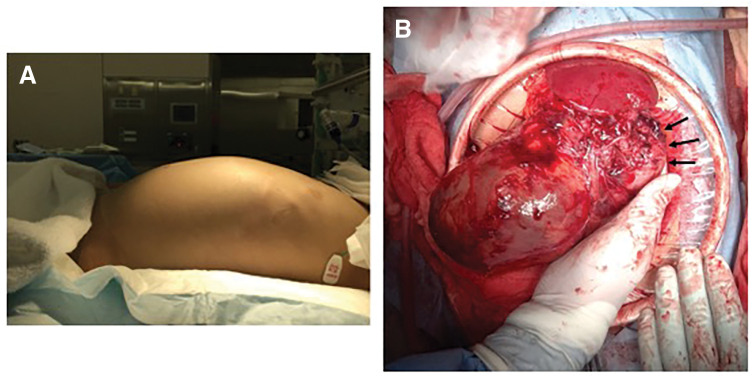
Intraoperative findings. (**A**) Clinical photograph showing abdominal distension prior to surgery. (**B**) The tumor capsule was ruptured near the inferior aspect of the spleen (black arrows).

A 4-year-old girl presented to a local clinic with fever and abdominal pain. During examination, a palpable abdominal mass was identified. Subsequent abdominal ultrasonography revealed a substantial tumor located at the upper pole of the left kidney. Therefore, the patient was referred to our hospital for further assessment of the mass. On admission, a hematological analysis indicated anemia with a hemoglobin level of 10.5 g/dL after transfusion of red blood cell concentrate at the referring hospital. Tumor markers were notably elevated; her neuron-specific enolase level exceeded 300.0 ng/mL (normal range: < 16.3 ng/mL). By contrast, urinary catecholamine metabolites remained within the normal limits, with a vanillylmandelic acid level of 5.8 μg/mg creatinine (Cre) and a homovanillic acid level of 21.5 μg/mg Cre. Contrast-enhanced abdominal CT revealed a heterogeneous 17 × 11 × 9.5-cm mass that caused compression of the left kidney. The kidney appeared deformed because of significant displacement and exhibited a beak sign suggestive of possible tumor invasion into the renal parenchyma. Lateral to the kidney, a low-density area encapsulated by a membrane suggestive of a hematoma was also identified (**Fig. 1A**). The central region of the tumor lacked enhancement, indicating necrosis or tumor degeneration. The left renal artery and vein were encased between the tumor and enlarged para-aortic lymph nodes, consistent with risk factors defined by imaging (**Fig. 1B**). Metaiodobenzylguanidine scintigraphy demonstrated uptake at the tumor periphery without evidence of distant metastases. PET CT revealed the absence of distant lesions. Based on the elevated tumor markers and imaging findings, a diagnosis of neuroblastoma was established. Owing to the presence of intratumoral hemorrhage and the associated high risk of further bleeding, biopsy was deemed unsafe and therefore postponed. The patient initially received treatment comprising 1 course of COG A3961 chemotherapy, including carboplatin and etoposide, for intermediate-risk neuroblastoma. On day 7, the patient exhibited significant abdominal distention and anemia with a hemoglobin level of 4.7 g/dL. A subsequent contrast-enhanced CT revealed increased ascites; thus, tumor rupture was suspected (**Fig. 1C**–**1D**). At the time of clinical deterioration associated with tumor rupture, no marked changes in vital signs were observed, and the patient did not fulfill the criteria for hemorrhagic shock. Laboratory tests showed a hemoglobin level of 4.7 g/dL, and a continuous furosemide infusion was required to maintain urine output. Consequently, emergency transarterial embolization (TAE) was performed targeting the suspected feeding arteries of the tumor, the left renal artery (**Fig. 2A**–**2C**), inferior phrenic artery, and adrenal branches of the celiac trunk (**Fig. 2D**–**2F**), despite the absence of a clearly identifiable bleeding source. Because multiple feeding arteries to the tumor originated from the renal artery, embolization of the renal artery was unavoidable. Hemorrhage caused by tumor rupture was controlled using TAE. Angiographic hemostasis was achieved in 2 h and 28 min. However, the patient’s intra-abdominal pressure increased from 14 to 20 mmHg after the procedure; during the subsequent 2 h, urine output was 0 mL (anuria), and the patient required endotracheal intubation with invasive mechanical ventilation (**Fig. 3A**). These findings were consistent with the development of ACS, prompting an emergency laparotomy on the same day. During a midline laparotomy, a substantial volume of hemorrhagic ascites was observed in the abdominal cavity. The neoplasm originated from the left adrenal gland and was accompanied by para-aortic lymphadenopathy encasing the renal hilum and displacing the kidney in a caudal direction. The tumor capsule was ruptured near the inferior aspect of the spleen (**Fig. 3B**). The renal artery and vein were identified and ligated after dissection through the enlarged lymph nodes, and the left ureter was subsequently transected. En bloc resection of the tumor and left kidney was performed. The operative duration was 4 h and 15 min, and total blood loss was 2968 mL in a 14.4 kg patient who received intraoperative transfusions of RCC 560 mL, FFP 480 mL, and platelet concentrate 10 units (200 mL). A histopathological examination confirmed neuroblastoma originating from the adrenal gland with invasion into the adjacent renal parenchyma. A molecular analysis revealed MYCN amplification. According to the International Neuroblastoma Risk Group,^6)^ this was a high-risk case.

After surgery, chylous ascites developed; however, it resolved by POD 11, thus allowing for drain removal. Chemotherapy utilizing the 05A3 protocol, which included cyclophosphamide, vincristine, pirarubicin, and cisplatin, was recommenced on day 16. The patient completed 11 cycles of chemotherapy, followed by autologous peripheral blood stem cell transplantation, proton beam therapy (30.6yE/17F), and 6 cycles of anti-GD2 antibody treatment. Retinoic acid therapy is currently being considered; however, it has not yet been approved for use in Japan. At 2 years postoperatively, complete remission has been maintained.

## DISCUSSION

This report presents a rare but severe case of MYCN-amplified neuroblastoma that resulted in tumor rupture and ACS soon after initiation of induction chemotherapy in a 4-year-old girl. Although hemorrhage that resulted from tumor rupture was controlled by TAE, ACS progression required emergency surgical intervention. Ultimately, a successful outcome was achieved through a multidisciplinary approach.

Tumor rupture in neuroblastoma is a rare but life-threatening complication, particularly during or after chemotherapy. Its pathophysiology involves a sudden increase in intratumoral pressure, progression of necrosis, and breakdown of the immature tumor vasculature.^4,7)^ MYCN-amplified neuroblastomas exhibit high proliferative activity and abnormal vascular structures, rendering them susceptible to hemorrhage and rupture after cytotoxic therapy.^2,8)^ Voglino et al. reported that among 44 patients with high-risk neuroblastoma, 4 (9%) experienced hemorrhagic complications, specifically hemothorax or intraperitoneal bleeding, within 2–7 days after initiating induction chemotherapy.^9)^ Our case involved a high-risk neuroblastoma, and chemotherapy-induced tumor rupture resulted in massive hemorrhagic ascites and ACS. Although rare, high-risk neuroblastoma can have a severe clinical course; therefore, a careful pretreatment assessment is essential. Imaging findings such as large tumor size, capsular tension, central necrosis, and invasion into adjacent organs or vessels may be indicators of potential rupture and should be closely evaluated.

A retrospective analysis of 47 cases of ruptured neuroblastoma identified a tumor diameter greater than 13.2 cm as a significant risk factor for rupture.^3)^ In our case, the tumor measured 17 × 11 × 9.5 cm, with the longest axis at 17 cm; therefore, it was categorized as high risk. However, because intratumoral hemorrhage occurred at presentation, the apparent tumor size may have been enlarged by hemorrhagic expansion; therefore, the dimensions before rupture may not have been accurately reflected. Although the applicability of the cutoff of 13.2 cm remains unclear, tumor enlargement clearly contributed to rupture. The tumor was also identified as an undifferentiated neuroblastoma with MYCN amplification, which is a recognized risk factor for rupture according to genetic pathology.

Intraperitoneal hemorrhage was suspected after the tumor ruptured; therefore, TAE was performed. TAE is a minimally invasive technique that achieves hemostasis; therefore, it has gained attention as an effective method of controlling bleeding in pediatric patients with solid tumors. However, while TAE is generally minimally invasive, it can be time-consuming; for patients in hemorrhagic shock, emergency laparotomy may be prioritized for rapid hemostasis. In our hemodynamically stable patient without shock, the bleeding source appeared embolization-amenable, and immediate surgical backup was available, supporting TAE as the first-line hemostatic approach. Yoshida et al. documented a pediatric case of a ruptured adrenal tumor that was successfully stabilized with TAE before surgery; therefore, TAE may be an effective bridge to surgical intervention for hemorrhagic solid tumors in pediatric patients.^10)^ The literature regarding TAE for tumor rupture with neuroblastoma is limited. This report describes the efficacy of TAE as an initial intervention for tumor rupture in patients with neuroblastoma.

In this case, tumor rupture resulted in rapid intraperitoneal hemorrhage and fluid accumulation. Although TAE achieved temporary hemostasis, sustained elevation of intra-abdominal pressure resulted in ACS. Minimally invasive decompressive procedures, such as percutaneous drainage or hematoma evacuation, are considered first-line interventions for ACS, particularly when tumors are near to major vessels or vital organs, because surgical resection is a high-risk procedure in such cases.^11,12)^ Several studies have reported the effectiveness of these approaches, including percutaneous decompression, which improved intra-abdominal pressure and organ function in pediatric patients.^4,13)^

In the present case, TAE included the left renal artery, which was identified as the major tumor-feeding vessel, thus rendering renal preservation unfeasible. Because nephrectomy was anticipated and the tumor appeared technically resectable, emergency surgery was deemed appropriate. The achievement of en bloc resection of the tumor and left kidney was fortuitous. In contrast, many ruptured neuroblastoma present with extensive involvement of major vessels and adjacent organs, which may preclude safe resection. In such scenarios, conservative management including percutaneous drainage may be the only feasible method of stabilizing intra-abdominal pressure. When definitive resection is not feasible due to extensive vascular involvement or physiological instability, damage control strategies, such as temporary decompression and staged surgery,^4)^ have been reported in rare pediatric cases.Our decision pathway similarly emphasized physiology-first stabilization, escalating from TAE to definitive surgery once ACS criteria were met.

Although surgical resection was definitive and successful in this case, it cannot be universally applied to all patients with tumor rupture and ACS. The decision to proceed with surgery must be carefully individualized by considering the anatomical extent, tumor biology, and hemodynamic stability. Further accumulation of cases and discussions is warranted to determine the optimal strategies for managing ACS in patients with high-risk neuroblastoma after TAE.

## CONCLUSIONS

Tumor rupture is a rare but life-threatening complication of high-risk MYCN-amplified neuroblastoma and ACS after induction chemotherapy. Despite hemostasis achieved with TAE, emergency tumor resection was necessary. This case emphasizes the need for pretreatment risk assessments and early surgical readiness of high-risk cases. Surgical resection can serve as a definitive treatment for ACS when anatomically feasible.

## DECLARATIONS

### Availability of data and materials

The datasets generated and/or analyzed during the current study are available from the corresponding author upon reasonable request.
